# A survey of clinical competence of new nurses working in emergency department in Iran: A descriptive, cross‐sectional study

**DOI:** 10.1002/nop2.579

**Published:** 2020-07-28

**Authors:** Behjat Vand Tamadoni, Shahla Shahbazi, Alehe Seyedrasooli, Neda Gilani, Leila Gholizadeh

**Affiliations:** ^1^ Department of Medical‐Surgical Nursing Faculty of Nursing and Midwifery Tabriz University of Medical Sciences Tabriz Iran; ^2^ Department of Medical‐Surgical Nursing Faculty of Nursing and Midwifery and Sina Hospital Tabriz University of Medical Sciences Tabriz Iran; ^3^ Department of Statistics and Epidemiology Faculty of Health Tabriz university of medical sciences Tabriz Iran; ^4^ Emergency Medicine Research Team Tabriz University of Medical Sciences Tabriz Iran; ^5^ IMPACCT Faculty of Health University of Technology Sydney Sydney NSW Australia

**Keywords:** competency, emergency department, emergency nursing, Iran, nurse competence, nursing

## Abstract

**Aims:**

This article reports on a study investigating the self‐assessed clinical competence of new nurses working in emergency departments.

**Design:**

A quantitative approach using descriptive cross‐sectional survey design was employed.

**Methods:**

The clinical competency of the participants was assessed using the Competency Inventory for Registered Nurse questionnaire, which contains the seven dimensions of clinical care, leadership, interpersonal relations, legal/ethical, professional development, teaching/coaching and critical thinking/research aptitude. Data from 115 new nurses employed in emergency departments of nine selected university hospitals in the northwest of Iran were collected by the Competency Inventory for Registered Nurse (CIRN) from December 2018–May 2019 and analysed.

**Results:**

The mean clinical competency for the total scale was 155.7 (*SD* 32.9), indicating a moderate competency. The most highly self‐rated competency was legal/ethical practice, and the least rated was critical thinking–research aptitude.

## 1 INTRODUCTION

Globally, the nursing profession is experiencing a shortage; this is particularly evident in highly specialized hospital units, such as emergency departments (Gorman, 2019; Schriver, Talmadge, Chuong, & Hedges, 2003). Consequently, newly graduated nurses are employed to work in emergency departments (Salonen, Kaunonen, Meretoja, & Tarkka, 2007). In such wards, that dealing with patients facing imminent life‐threatening situations is a common feature, the clinical competence of care providers is a concern. The competency of nursing staff in providing emergency care is a critical factor in patient safety. Further, the quality of services provided in the ED is considered a hospital performance indicator (Tourani et al., 2019). Therefore, nurses and other multidisciplinary team members must be competent and work collaboratively to improve patient and healthcare outcomes (Holanda, Marra, & Cunha, 2019). Considering the complexity and dynamic nature of the ED, ensuring the clinical competency of nursing staff, in particular new nurses, is prominent. Nursing competency has been defined by the International Council of Nurses (1997, p. 44) as: “a level of performance demonstrating the effective application of knowledge, skills and judgments” (International Council of Nurses, 1997).

## 2 BACKGROUND

Consistent with many other countries in the world, nursing shortage is a challenging issue for the healthcare system of Iran. According to the national code of ethics, nurses are expected to be competent, have sufficient knowledge and skills to provide safe and quality care independently and assume accountability for their actions (Zahedi et al., 2013). Globally, newly graduated nurses perceive low competency to work independently, requiring support and mentoring within the first a few months of their practice as a registered nurse (Baumann, Crea‐Arsenio, Hunsberger, Fleming‐Carroll, & Keatings, 2019; Spence Laschinger et al., 2019). In Iran, nurses should have at least three years of work experience as a nurse to be able to work in the EDs. However, due to the nurse shortage, this employment requirement is not always met, jeopardizing patient safety in the EDs. Indeed, the phenomenon of new nurses commencing their professional career in an emergency department is relatively new in the nursing world (Kary, 2012).

The clinical competence of nurses is a widely studied topic globally (Feliciano, Boshra, Mejia, Feliciano, & Maniago, 2019; Flinkman et al., 2017; Hansen‐Salie & Martin, 2014; Hengstberger‐Sims et al., 2008; Istomina et al., 2011; Liu, Yin, Ma, Lo, & Zeng, 2009; Liu & Aungsuroch, 2018; Salonen et al., 2007; Tam, 2015; Tsutsumi & Sekido, 2015) and in Iran (Hassankhani, Hasanzadeh, Powers, Zadeh, & Rajaie, 2017; Mirlashari, Qommi, Nariman, Bahrani, & Begjani, 2016). However, the clinical competence of new nurses in the EDs is a research gap in the literature. The current study aimed to answer the following question: What is the self‐assessed clinical competence of new nurses working in the EDs in Iran.

## 3 THE STUDY

### 3.1 Design

This was a descriptive cross‐sectional survey design study.

### 3.2 Method

Nurses with equal or less than 3 years of work experience in the EDs of nine selected university hospitals in the northwest of Iran were invited to participate in the study (*N* = 132), of whom two nurses declined to participate in the study, for reasons, such as having a busy workload and being tired. Participant recruitment took place using convenience sampling.

Overall, 130 new emergency nurses completed the survey, 15 questionnaires were removed due to incomplete data, leaving 115 questionnaires for analysis. Data were collected by a paper–pencil questionnaire from December 2018–May 2019.

The study tool consisted of a self‐administered survey containing a section on demographic and professional characteristics and the Competency Inventory for Registered Nurses (CIRN) questionnaire (Liu et al., 2009). The CIRN is a standardized questionnaire consisted of 55 items and the seven dimensions of clinical care, leadership, interpersonal relations, legal/ethical, professional development, teaching/coaching and critical thinking/research aptitude. The CIRN uses a 5‐point Likert scale ranging from 0 (not competent at all)–4 (very competent). The scores for the total scale can range from 0–220. The Content Validity Index of the CIRN was 0.85 in the original study (Liu et al., 2009). It has been translated into Persian and psychometrically tested in Iran on a sample of registered nurses (Ghasemi, Janani, Dehghan Nayeri, & Negarandeh, 2014). The Cronbach's alpha coefficient of the Persian version of the CIRN in the current study was 0.968 for the total questionnaire and the scores ranged between 0.960–0.966 for the seven competency domains. The completion of the survey took an average of 25 min. Permission to apply the Competency Inventory for Registered Nurse (CIRN) and to make any sort of modifications according to our practical context was gained from Dr. Liu by Email contact (Sat, 23 Sep 2017 at 12:45 pm).

### 3.3 Analysis

The data were analysed using SPSS software (IBM SPSS software (version 24; SPSS)) with the assistance of the research team statistician. Descriptive statistics, including frequency distribution, mean, standard deviation, range were used to summarize the data.

### 3.4 Ethics

Before collecting the data, the Regional Research Ethics Committee of Tabriz University of Medical Sciences issued permission for conducting the study (IR.TBZMED.REC.1397.395). All participants received oral and written information about the study and its objectives and were informed that the participation was voluntary, and they were free to withdraw from the study at any time. Those who were interested in participating in the study signed a consent form. The participants' confidentiality was ensured throughout the research process.

## 4 RESULTS

Table 1 summarizes the sociodemographic and professional characteristics of the participants. The participants' age ranged between 21–50 years, with a mean of 29.47 (*SD* 5.92). Most participants were female (64.8%), married (66.1%) and had a bachelor degree in nursing (94.8%). More than half of the participants (57.45) had less than five years work experience as a registered nurse, 21.7% commenced their first nursing job in the EDs and 29.6% had <1 year of work experience in the ED (Table 1).

**TABLE 1:** Sociodemographic and professional characteristics of the participants (*N* = 115)

Variables	Frequency (%)
Age (years)	
<30	70 (60.9)
30–40	37 (32.2)
≥40	8 (7.0)
Gender	
Male	37 (35.2)
Female	68 (64.8)
Education	
Bachelor of Science	109 (94.8)
Master of Science	6 (5.2)
Marital status	
Married	76 (66.1)
Single	38 (33.0)
Separated	1 (0.9)
Experience in the emergency department (years)	
<1	34 (29.6)
1–2	44 (38.3)
2–3	37 (32.2)
Work experience in other wards	
Yes	90 (78.3)
No	25 (21.7)
Total work experience as an RN (years)	
<5	66 (57.4)
5–10	30 (26.1)
10–15	10 (8.7)
15–20	5 (4.3)
≥20	4 (3.5)

### 4.1 Self‐assessed clinical competence

The total mean score for the CIRN was 155.7, with a standard deviation of 32.9, indicating an overall moderate competency. Slightly above half of the participants (53%) self‐rated their competency level as moderate. The normalized mean scores in the seven competency domains ranged from 66.6–76.6. The most highly rated clinical competency domain was related to legal/ethical practice (76.6), followed by leadership (72.0), interpersonal relation (70.5) and clinical care (70.4). The least highly rated domains included critical thinking–research aptitude (66.6) and teaching–coaching (66.6) (Table 2).

**TABLE 2:** Emergency new nurses' self‐assessed level of clinical competence (*N* = 115)

The CIRN domains	Number of items	Min	Max	Mean (*SD*)	95% CI	Normalized Mean (*SD*)
Clinical care	10	13	40	28.1 (6.3)	27.0–29.3	70.4 (15.8)
Leadership dimension	9	10	36	25.9 (5.7)	24.9–26.9	72.0 (15.9)
Interpersonal relation	8	10	32	22.6 (0.5)	21.6–23.5	70.5(15.7)
Legal/ethical practice	**8**	**9**	**32**	**24.5 (5.4)**	**23.5–25.5**	**76.6 (16.9** **)**
Professional development	6	7	24	16.6 (3.8)	15.9–17.3	69.3 (16.0)
Teaching–coaching	6	5	24	16.5 (4.1)	15.7–17.3	66.6 (16.5)
Critical thinking–research aptitude	**8**	**7**	**32**	**21.3 (5.3)**	**20.3–22.3**	**66.6 (16.5)**
Overall CIRN	55	70	218	155.7 (32.9)	149.6–161.7	

Abbreviations: CIRN, Competency Inventory for Registered Nurse; *SD*, standard deviation.

Bold values indicate to highlight the most highly self‐rated competency and the least rated competency.

## 5 DISCUSSION

In this study, nurses self‐rated their overall competence at a moderate level. No prior study has been carried out in Iran to investigate the clinical competence of new nurses in the EDs. In a study by Hassankhani et al. (2017), Iranian emergency nurses, including both new and experienced, self‐rated their competency level as good (Hassankhani et al., 2017). Mirlashari et al. (2016) studied the clinical competence of Iranian nurses employed in the neonatal intensive care units and reported that 65.8% of these nurses had a moderate clinical competency (Mirlashari et al., 2016), as opposed to 53% in our study. The higher percentage of nurses with a moderate clinical competency level in Mirlashari et al.'s (2016) study can be due to recruiting both highly experienced and less experienced nurses (Mirlashari et al., 2016). Outside Iran, nurses in different clinical settings often self‐assess their competence as good or high. In Salonen et al.'s (2007) study, registered nurses with less than three years of work experience and employed in intensive and emergency settings in Finland rated their competence as good (Salonen et al., 2007). A similar study conducted in South Africa (Hansen‐Salie & Martin, 2014) reported the self‐assessed clinical competence of new nurses employed in different hospital wards as high (Hansen‐Salie & Martin, 2014). A study conducted on 270 Lithuanian registered nurses working with patients after abdominal surgery also concluded that the nurses overall self‐evaluated their competency level as high. This study included both new and experienced nurses.

The relatively lower self‐rated competence in our study can be partially explained by the lower work experience of the participating nurses. It is well known that the length of work experience is associated with competence development (Aqtash et al., 2017; Meretoja, Numminen, Isoaho, & Leino‐Kilpi, 2015; Rush, Janke, Duchscher, Phillips, & Kaur, 2019). This finding is confirmed in Hassankhani et al.'s (2017) study in that a combined cohort of new and experienced nurses working in the EDs in Iran demonstrated a higher level of competency (Hassankhani et al., 2017). Also, there is a relationship between perceived competence and frequency of use of a specific competence (Aqtash et al., 2017; Hengstberger‐Sims et al., 2008).

In the current study, nurses rated themselves highly competent in providing legal/ethical practice, leadership, interpersonal relation and clinical care, but lowly in competency domains related to critical thinking/research aptitude and teaching/coaching. New nurses working in the EDs may not have adequate opportunities to practise some competency domains, such as teaching and coaching, or advancing nursing research through research. Similar to our findings, other studies have consistently reported a lower level of perceived competency by nurses in teaching–coaching and critical thinking/research aptitude (Feliciano et al., 2019; Hansen‐Salie & Martin, 2014; Istomina et al., 2011; Mirlashari et al., 2016).

Nurses' competency in teaching and coaching may be improved by providing opportunities to be engaged in patient education and mentoring and precipitating of novice nurses (Liu et al., 2009). Education is an essential part of nursing's role involving individuals and groups, patients and coworkers. Nursing curriculums should ensure that nurse students are given adequate opportunities to learn and practice effective patient education and presentation skills.

In addition, nurses should be supported to be engaged in research projects to develop competency in advancing the nursing profession through the conduction of high‐quality research. Training nurses in evidence‐based practice skills can enhance the uptake of research findings in clinical settings and facilities the implementation of evidence‐based practice (Newhouse, Dearholt, Poe, Pugh, & White, 2007).

In our study, for 21.7% of the participants, the ED was the initial entry site. Given that nurses' the clinical competency takes time to develop, employing novice nurses in the EDs may impose risk to patient safety. The ED is a fast‐paced dynamic environment that demands high critical thinking ability, clinical skills, prioritizing and communication (Buerhaus, Donelan, Ulrich, & Norman, 2005; Fero, Witsberger, Wesmiller, Zullo, & Hoffman, 2009). The complexity and diversity of patient care needs create significant competence challenges for emergency nurses and they need to be aware of the impact of optimal competence on patient care outcomes (Meretoja & Koponen, 2012). Patient safety may be compromised if a nurse cannot provide clinically competent care (Fero et al., 2009). For example, the critical thinking ability of a nurse can directly affect patient safety. Nurses must have the skills to recognize variations in patient conditions, perform independent nursing interventions, anticipate orders and prioritize care based on the circumstances. These actions require critical thinking ability, advanced problem‐solving skills and the ability to communicate clearly (Buerhaus et al., 2005; Fero et al., 2009). The new nurse preceptorship programs provide novice nurses with opportunities to enhance competence in different domains, including critical thinking and clinical skills (Gorman, 2019; Ke, Kuo, & Hung, 2017; Rush et al., 2019). Strategies such as in‐services and problem‐solving workshops may also improve the critical thinking skills of beginner nurses (López et al., 2020).

### 5.1 Limitations

The sample size of this study is small, yet, the participants were recruited from the emergency departments of nine teaching hospitals. In addition, subjectivity and response bias are inherent challenges in self‐assessment of competence (Meretoja & Koponen, 2012).

## 6 CONCLUSION

New nurses, working in the EDs, in Iran self‐assessed their competence as moderate. The results indicate the need for improving new nurses' competence in the EDs, with a particular focus on their teaching/coaching and critical thinking/research aptitude skills. This is the first report on the competence of new nurses working in the EDs.

### 6.1 Recommendations for practice and further research

The findings of this study provide implications for nursing administration, nursing practice and nursing education. The findings provide evidence to nursing mangers on the level of competency perceived by new nurses in the EDs and the potential patient safety issues. New nurses may be provided with an opportunity to develop their competency in a slow‐paced care setting, before working in the EDs. Ensuring appropriate skill mix in each working shift may help improve patient safety and facilitate peer learning. Nurse managers can also support new nurses by providing preceptorship, mentorship and supervision. The knowledge gained can help new nurse preceptors identify the learning needs of novice nurses and develop training programs to target their specific learning needs. The opportunities to practise and master nursing skills can increase nurses' perceived competency (Aqtash et al., 2017; Meretoja et al., 2015; Rush et al., 2019).

In addition, encouraging nurses to self‐assess their competence can increase their awareness of the competency areas lacking and the impact of this on patient safety. This study and other research suggest that nursing curriculums should focus on developing the teaching/education skills of nursing students and introducing them to research and evidence‐based practice skills. Finally, further research on big samples is required in Iran and other countries to validate the results of the present research.

## CONFLICT OF INTEREST

Authors declare no conflict of interest.

## AUTHOR CONTRIBUTIONS

BVT, SS, AS and NG: Study design; BVT and SS: Acquisition of data; NG and BVT: Analysis and interpretation of data; SS, BVT and NG: Drafting of the article; LG and SS: Critical revision of the manuscript for important intellectual content.
